# Cognitive Distortions in Relation to Plasma Cortisol and Oxytocin Levels in Major Depressive Disorder

**DOI:** 10.3389/fpsyt.2019.00971

**Published:** 2020-01-22

**Authors:** Susan Jennifer Thomas, Theresa Larkin

**Affiliations:** Faculty of Science, Medicine and Health and Illawarra Health and Medical Research Institute, University of Wollongong, Wollongong, NSW, Australia

**Keywords:** cognitive distortions, cortisol, oxytocin, major depressive disorder, stress

## Abstract

Negative thinking is prominent in major depressive disorder (MDD). Cognitive models propose that negative thinking influences the hypothalamus-pituitary-adrenal axis and cortisol release. Oxytocin is also linked to MDD, social and affective processing, and stress buffering. Little research has examined direct relationships between negative cognitions, cortisol, and oxytocin.

**Methods:**

Sixty-one unmedicated participants meeting DSM-5 criteria for MDD and 60 healthy controls completed measures of psychopathology, stress, and cognitions. Plasma samples were analyzed for cortisol and oxytocin. Between-group analyses of variance were conducted along with correlational, regression and mediation analyses.

**Results:**

Depressed participants reported greater frequency and believability of negative thoughts than controls. Cortisol levels were positively, and oxytocin inversely, correlated with negative thinking. Cortisol and negative thinking accounted for unique variance in depression, and the relationship between stress and cortisol depended on the extent of negative cognitions.

**Conclusions:**

The results support long-standing cognitive models which propose that negative thoughts are important in the relationship between stress and cortisol levels.

## Introduction

Cortisol dysregulation (1, 2) and pessimistic thinking styles (3, 4) are both independently strongly linked to depression. Additionally, the oxytocinergic system is involved in affective and social processing (5, 6), stress buffering (7), and is also related to depressive symptoms (8). While cortisol, oxytocin, and negative thinking are all linked to depression, there is still little research examining direct relationships between these factors.

The development, severity, and course of MDD are closely related to psychosocial stress. A key mechanism linking stress and MDD is altered hypothalamic-pituitary-adrenal (HPA) functioning. The HPA system allows organisms to adapt to physical and psychosocial changes in their environments. In humans, perceived stress activates the central nervous system (CNS), which stimulates the release of corticotropin releasing hormone (CRH) from the hypothalamus, adrenal corticotrophic hormone (ACTH) from the anterior pituitary, and cortisol from the adrenal cortex. Cortisol mobilises glucose stores, to provide the organism with energy. Cortisol affects substructures of the hippocampus and the amygdala, reinforcing emotional encoding of the stressful situation and potentially altering the organism's future responses to similar stressors. Adaptive cortisol responses are characterized by a quick rise in response to stress and a rapid decline after the challenge has passed, achieved by negative feedback mechanisms in the hippocampus (reviewed in 9). HPA axis function is influenced both by genetics and by early adverse experiences. There is well-established evidence from animal studies that psychosocial stress, such as early separation of non-human primates from their mothers, can lead to persistent disturbances in the regulation of cortisol secretion (10). Depression is associated with chronic stress and hyperactivity of the hypothalamic-pituitary-adrenal (HPA) axis, and a significant percentage of depressed individuals have increased cortisol levels (1, 2). Exposure to stress is therefore a vulnerability factor to developing depression, which could cause impairments to HPA functioning (11, 12).

Stress can be defined as a psychophysiological response to real or perceived pressures in the environment, including danger (13). While adverse life events and stressors are risk factors for developing MDD, it is not purely the power of environmental demands which determines their emotional impact, but the individual's psychological appraisals of their ability to cope (14). Patterns of negative thinking are recognized as a hallmark of depression (15) and are included in the diagnostic criteria for major depressive disorder (16). In Beck's cognitive model of depression (3), early adverse events, along with genetic factors, contribute to three characteristic aspects of thinking which perpetuate depression; the cognitive triad, schemas, and cognitive errors. The cognitive triad consists of automatic negative thoughts about the self (e.g. *I am worthless*), the world (e.g. *there are insurmountable obstacles preventing me from reaching goals*), and the future (e.g. *this suffering will continue indefinitely*). Schemas are long-term, stable cognitive patterns of organizing and interpreting information, which reduce mental effort by guiding behavior in many routine situations. However, people with depression will often hold negative schemas following aversive early experiences such as criticism or abuse. These dysfunctional attitudes remain latent but are activated through stress or biological factors, leading to information being interpreted in a pessimistic way. This in turn leads to a number of characteristic cognitive distortions, which have further negative impacts on mood and behavior (3).

Cognitive distortions include negative filtering, where only aspects of reality which conform to negative beliefs are attended to, and dichotomous thinking, viewing situations in extreme terms, where even small defects are painful. These types of thinking represent primitive thinking styles (3) which may have evolved as fast-track defensive forms of thinking that are adaptive to survival in times of threat (17). Through negatively biased information-processing, however, negative thinking can become automatic, serving to maintain and rekindle depressive episodes (3, 4, 18). Thus cognitive approaches to depression postulate biases in automatic, associative forms of thinking and a failure to correct these through slower, more reflective thought processes (19).

More recently, cognitive models have evolved to integrate neurobiological, particularly brain imaging, data (4). For example, cognitive biases in depression have been linked to maladaptive bottom-up patterns of activation initiated in subcortical brain regions, accompanied by impairment in the top-down cognitive control of subcortical emotional activity by higher-level cortical regions (18, 20). Such models propose that a hypersensitive amygdala and hypoactive prefrontal regions are associated with impairments in the ability to think rationally in depression (4). This is supported by indications that when attending to emotional stimuli, individuals with depression show more intense and longer-lasting amygdala reactivity (21), along with impaired attentional disengagement which is correlated with decreased activity in the right ventro- and dorso-lateral-pre-frontal cortical areas compared to healthy controls (22, 23). Beck proposed a hypothetical pathway starting with genetic vulnerability, leading to excessive amygdala reactivity and attentional deployment to emotional stimuli, and negative information-processing biases resulting in cognitive distortions and negative schemata. These negative interpretations of experience trigger the hypothalamus-pituitary-adrenal (HPA) axis more frequently or intensely, leading to cortisol responses which may affect the serotonergic system and lead to depression (4).

Over-reactions to stress, and hypercortisolemia seen in depression have been proposed to be mediated by cognitive distortions (4). Cognitive appraisals may modulate emotional responses through connections from the prefrontal cortex to limbic areas including the amygdala and hippocampus, which are connected to the hypothalamus (24). In healthy participants, the HPA axis can be reliably activated by psychological stress (24). There is little research examining relationships between patterns of negative thinking and cortisol levels in MDD. One early study examined relationships between salivary cortisol and negative cognition in depression over time. There were indications that changes in negative automatic thoughts preceded changes in cortisol, however the results were ambiguous, and only eight participants completed the study (25).

While several lines of evidence give reason to believe that negative cognitions, stress, and cortisol are interlinked in MDD, there is surprisingly little direct investigation of relationships between these key variables. Depressive symptoms, stress, and negative cognitions are considered to be distinct concepts which can be reliably discriminated by psychometric tools, although they may be correlated due to shared aetiological factors such as personality and environmental factors (26). For example, the frequency and intensity of negative thoughts can be measured with the Automatic Thoughts Questionnaire (ATQ; 27). Negative cognitions correlate with measures of depression, however the ATQ is not synonymous with depression, as depressive symptoms include additional affective, somatic, and cognitive factors (16), and cognitive distortions are also elevated in people with other disorders such as anxiety, relative to controls (28). The depression anxiety and stress scales (DASS) discriminate between depression and stress as distinct and non-overlapping concepts, with stress encompassing symptoms of a state of persistent arousal and tension with a low threshold for becoming upset or frustrated (26). We therefore sought to measure these distinct but related concepts in relation to cortisol and to evaluate Beck's (4) theoretical model of relationships between them.

The oxytocinergic system is involved in a range of complex social behaviors in humans and plays a key role in affective and social processing and behavior (5, 6). There is evidence that oxytocin may reduce anxiety in response to stressors by down-regulating HPA axis activity, reducing amygdala reactivity (6) and dampening stress responses (7, 29, 30). There are relatively few studies on plasma oxytocin levels in depression, however there is some evidence that oxytocin levels are inversely correlated with depressive symptoms (8, 31), however a small study (*n =* 11) found higher oxytocin levels in depressed than control participants (32). It has been hypothesized that some symptoms of depression including social withdrawal, reduced appetite, and cognitive impairment may reflect central oxytocin alterations (33). Additionally, animal studies indicate that the oxytocinergic and HPA systems show complex independent as well as potentially synchronized activations which are not fully understood (e.g. 34). Investigating markers of activity of both the HPA and oxytocinergic neuroendocrine systems in relation to stress, depression, and cognitive distortions may help to give a better understanding of their relative importance in MDD. One study of healthy individuals investigated whether rumination might function as a moderator between depressive symptoms and oxytocin (35). It was found that high depressive symptoms were negatively associated with oxytocin concentrations at high but not low levels of maladaptive thinking (35), however the study was limited by an all-male, non-clinical sample. To our knowledge, no previous studies have investigated oxytocin levels in relation to negative automatic thoughts in MDD. Examining the neurobiology of negative cognitions may inform integrated cognitive-biological models of depression, and cognitive-behavioral interventions for emotional disorders (36).

## Aims and Hypotheses

The current study investigated the link between negative cognitions, cortisol, and oxytocin in individuals with MDD. We recruited individuals meeting diagnostic criteria for MDD and controls with no history of MDD and collected psychometric measures and blood samples. Specifically, we aimed to:

1. Investigate differences in the frequency and strength of belief in negative cognitions between a group with MDD and controls. It was predicted that participants with MDD would have higher frequency and greater strength of belief in negative cognitions.
2. Investigate relationships between negative cognitions, cortisol, and oxytocin. It was predicted that negative cognitions would be associated with higher cortisol levels and lower oxytocin levels.
3. Ascertain whether negative cognitions, cortisol, and oxytocin each account for unique variance in symptoms of major depressive disorder. It was predicted that negative cognitions and cortisol levels would positively predict, and oxytocin concentrations would inversely predict, depressive symptoms.
4. Evaluate aspects of Beck's (4) hypothesis that negative cognitions mediate relationships between stress and cortisol. In line with Beck's hypothesis, it was predicted that the relationship between stress levels and cortisol would depend on the extent of negative cognitions.

## Methods

### Participants

The data were collected as part of a larger study of MDD, and the protocol as well as mean cortisol, oxytocin, and demographic data for this cohort have previously been reported in the context of help-seeking (37) and quality of life (38). This study was conducted in accordance with the recommendations of the Australian National Statement for the Ethical Conduct of Research. The protocol was approved by the joint health district and university ethics committee (protocol number 2012409). All participants gave written informed consent.

Sixty-three adults were recruited who met the DSM-5 diagnostic criteria for MDD, along with 60 healthy controls. Participants were recruited through advertisements in local media and at the university. All participants were screened beforehand to ensure that depressed participants likely met DSM-5 criteria for MDD and had not taken anti-depressant medication for at least two months, and that healthy controls had no mental disorders or other exclusion criteria. Depressed participants were also interviewed on arrival at the clinical trials unit by an experienced clinical psychologist to confirm that they met diagnostic criteria for major depressive disorder using the Mini International Neuropsychiatric Interview (39, 40) version 7.0.2 for DSM-5, and to ascertain treatment history. All participants were asked about any past or present mental health problems. Exclusion criteria across groups included any neurological disorders such as epilepsy, head injuries or degenerative disorders as well as substance use disorders, and use of corticosteroid medication within the last two months.

### Measures

All participants completed the following measures: The Depression, Anxiety and Stress Scales-21 (41), an overall measure of psychological distress during the past seven days, and incorporates *Depression*, *Anxiety,* and *Stress* subscales. The Automatic Thoughts Questionnaire (27) is a 30-item questionnaire which measures the frequency of occurrence of automatic negative thoughts (negative self-statements) and believability and strength of belief in these statements if they occur. Respondents indicate how frequently each thought occurred during the past week (1 = *not at all*, 5 = *all the time*), with higher scores indicating greater frequency of negative automatic thoughts. The ATQ has four subscales which reflect aspects of these automatic thoughts: *Personal maladjustment and desire for change* (PMDC), *Negative self-concepts and negative expectations* (NSNE), *Low self-esteem* (LSE), and *Helplessness*. The Total score is the sum of questions 1 to 30 and indicates the frequency with which these thoughts occur. An additional ATQ scale assesses the strength of belief (1 = *not at all*, 5 = *totally*) in negative thoughts. Because the ATQ assesses thought processes during the past week, it provides a measure of the state of recent thinking patterns rather than long-term traits. Additional psychometric and biometric data were also collected as part of a larger study of MDD.

### Blood Collection and Processing

All blood samples were taken between 9 and 11 am. Each participant gave one blood sample, and collection times were matched between groups. A phlebotomist drew 8 ml of blood from a vein in the cubital fossa into tubes with EDTA which were immediately placed on ice. Aprotinin was added (25 μl/ml blood) within 5 minutes of collection. Within 20 minutes of collection, blood was centrifuged at 2800 rpm and 4°C for 10 minutes, after which plasma was aliquoted and stored at −80°C until analysis. Plasma cortisol and oxytocin were measured using a standard enzyme linked immunosorbent assay (ELISA) method with detection at 450 nm. Samples and standards were run in triplicate. The cortisol and oxytocin assays had a sensitivity of 2.44 ng/ml and 15 pg/ml, respectively, with intra-assay and inter-assay variance of less than 9% and 10%, respectively for cortisol, and less than 14% and 20%, respectively for oxytocin.

### Statistical Analyses

Prior to analysis, the variables were examined for skewness and kurtosis and transformed where necessary to better approximate normal distributions. Z-tests were applied to test for normality using skewness and kurtosis. Z-scores were obtained by dividing the skew values or excess kurtosis by their standard errors, using a critical value of 3.29 for medium-sized samples (n *=* 50–300), at which the null hypothesis would be rejected and the distribution considered non-normal (42, 43).

Two-way group (MDD, control) by Sex (male, female) ANOVAs were performed to examine the effects and interactions of clinical status and sex on each study variable. Pearson correlations were used to assess relationships between the variables. We conducted preliminary correlational analyses in each group separately to establish that the correlations between variables were in the same directions across groups. Having confirmed this, the main correlational analyses were conducted across all participants, as the variables were conceptualized to occur in a continuum in the population, and power analyses using G*Power 3.1.9.3 (44) indicated that separate group analyses would be underpowered, as in order to detect a medium effect size (*p* = 0.3), with an alpha of 0.05, and a power of 0.8, a sample size of 84 people is needed.

A multiple regression was performed to ascertain whether negative cognition, cortisol, and oxytocin were all significant unique predictors of depression scores, while controlling for age and sex. We tested the extent to which negative thinking (ATQ Total score), cortisol, and oxytocin levels account for unique variance in DASS depression score after accounting for demographic variables.

Next we conducted a mediation analysis to assess the indirect relationship between stress (measured by the DASS Stress score) and cortisol levels through a proposed mediator (negative cognitions, measured by the ATQ Total score) using the Process method (45) and the recommendations of Baron and Kenny (46). Age was included as a covariate in all regression models. We hypothesized that stress would be a risk factor for higher cortisol levels, and that cortisol levels would depend on the extent of negative cognitions. We tested the following predictions:

1. Stress levels would be related to cortisol levels (*path c*).
2. Stress levels would be related to negative cognitions (*path a*).
3. When both stress and negative cognitions are entered simultaneously into the regression equation as predictors of cortisol, negative cognitions would be related to cortisol (*path b*) however stress would no longer predict, or would be lessened in predicting, cortisol levels (*path c′*).

If results from all three equations were satisfied according to Baron and Kenny's (46) criteria, it would suggest that negative cognitions affect the relationship between stress and cortisol levels. Because the indirect effect may not be normally distributed, the 95% confidence interval (CI) was derived from bootstrap resampling (50,000). If the CI produced does not cross zero then criteria for mediation have been met (47).

## Results

Participant characteristics and between-group analyses are presented in **Table 1**. Blood collection was unsuccessful for two participants with MDD, hence the final data set included 61 participants with MDD and 60 controls. Eight participants with MDD reported a history of anxiety, one reported a previous adjustment disorder and one had previous post-traumatic stress symptoms. The MDD and control groups did not differ significantly on age. The male: female ratio was 25:35 in the control group and 26:35 in the MDD group. Chronbach's alpha indicated good-excellent reliability of the psychometric scales (**Table 1**). For the psychometric scales, absolute z-scores for skewness (1.87 to 2.17) and kurtosis (−1.1 to −2.5) were below the acceptable critical value of 3.29, indicating acceptable normality. Both hormones were non-normally distributed and therefore were transformed prior to further analyses. Cortisol was positively skewed, (kurtosis: 5.3, SE.44; skewness: 1.7, SE 0.22) which improved with log transformation (kurtosis: −0.25, SE 0.44; skewness: −0.35, SE 0.22). Oxytocin was also positively skewed (kurtosis: 0.46, SE 0.43; skewness: 0.92, SE 0.22) which improved with square-root transformation (kurtosis: −0.44, SE 0.43; skewness: −0.23, SE 0.22). Two-way ANOVAs with diagnostic Group (MDD, control) and sex (female, male) as between-group factors indicated that there were no significant differences between males and females, and no Sex by Group interactions for any of the study variables (*P* > 0.05 in all cases), hence all further analyses were run with data from both sexes combined. The MDD group scored significantly higher than controls on the Total ATQ score and each ATQ subscale. The MDD group had higher Depression and Stress scores, higher cortisol levels, and lower oxytocin levels than controls (**Table 1**).

**Table 1:** Descriptive data and analyses of variance between participants with MDD (n = 61) and controls (n = 60) for demographic, hormonal, and psychometric study variables.

Variable		MDD	Control				
		Mean (SD)	Mean (SD)	Chronbach's alpha	*F*	*p*	*η* ^2^ *_p_*
Age	Years	31.8 (14.63)	32.3 (10.98)		0.04	0.85	0.00
Cortisol	nmol/L	252.29 (102.63)	106.65 (64.53)		86.98	<0.001	0.42
Oxytocin	pg/ml	158 (140)	261.7 (158.97)		14.68	<0.001	0.11
DASS	Depression	22.98 (9.42)	6.2 (7.4)	0.94	118.42	<0.001	0.50
	Stress	21.51 (9.49)	10.30 (7.97)	0.89	49.39	<0.001	0.29
ATQ	Personal maladjustment and desire for change	17.44 (5.52)	9.35 (4.53)	0.91	77.63	<0.001	0.40
	Negative self-concepts and negative expectations	21.13 (8.23)	10.83 (4.93)	0.94	69.36	<0.001	0.37
	Low self-esteem	5.52 (2.94)	2.75 (1.57)	0.90	41.83	<0.001	0.26
	Helplessness	5.77 (2.18)	3.15 (2.08)	0.73	45.74	<0.001	0.28
	Total Strength of negative beliefs	89.38 (29.23)	55.88 (35.09)	0.98	32.6	<0.001	0.22
	ATQ Total frequency of negative beliefs	91.43 (30.88)	49.45 (21.35)	0.98	69.16	<0.001	0.38

DASS, Depression, Anxiety, and Stress Scales; ATQ, Automatic Thoughts Questionnaire.

Preliminary correlational analyses indicated that the direction of relationships between the variables was similar across groups. Correlations are therefore reported across both groups (**Table 2**). Cortisol was positively correlated with depression, anxiety, stress, and all scales of the ATQ. Oxytocin was inversely correlated with depression, stress, ATQ helplessness, personal maladjustment and desire for change, negative self-concepts and negative expectations, total strength of negative beliefs, and total frequency of negative beliefs. Depression correlated positively with anxiety, stress, and all measures of the ATQ. Additionally, the subscales of the ATQ were correlated with each other.

**Table 2:** Pearson's Correlations for Study Variables.

Variable	1	2	3	4	5	6	7	8	9	10
1. Cortisol										
2. Oxytocin	0.07									
3. DASS Depression	0.53**	−0.26**								
4. DASS Anxiety	0.39**	−0.18	0.74**							
5. DASS Stress	0.42**	−0.23**	0.78**	0.78**						
6. ATQ Personal maladjustment and desire for change (PMDC)	0.41**	−0.28**	0.82**	0.65**	0.74**					
7. ATQ Negative self-concepts and negative expectations (NSNE)	0.43**	−0.27**	0.85**	0.69**	0.74**	0.89**				
8. ATQ Low self-esteem (LSE)	0.40**	−0.17	0.76**	0.63**	0.64**	0.8**	0.86**			
9. ATQ Helplessness	0.39**	−0.23**	0.77**	0.66**	0.76**	0.81**	0.84**	0.71**		
10. ATQ Total strength of negative beliefs	0.34**	−0.18*	0.67**	0.55**	0.58**	0.73**	0.74**	0.68**	0.66**	
11. ATQ Total frequency of negative beliefs	0.44**	−0.27**	0.88**	0.71**	0.78**	0.95**	0.97**	0.88**	0.87**	0.77*

DASS, Depression, Anxiety, and Stress Scales; ATQ, Automatic Thoughts Questionnaire.

**Correlation is significant at the 0.01 level (two-tailed).

*Correlation is significant at the 0.05 level (two-tailed).

Tolerance was greater than 0.10 and variance inflation factor values were all below 3, indicating an absence of multicollinearity. Mahalanobis distance test identified seven multivariate outliers. The regression analyses were run both with and without the outliers, and the results were equivalent, hence it was decided to retain the outliers, and results are reported with the full data set. For the regression analyses, the assumptions of normality, linearity, and homoscedasticity of residuals were considered to be satisfied based on the inspection of plots of standardized residuals and predicted values.

Results of the multiple regression analysis investigating the relative contribution of negative cognitions, cortisol, and oxytocin as independent predictors of DASS depression are shown in **Table 3**. Age, sex, ATQ total score, cortisol, and oxytocin levels were entered as independent variables, and collectively the predictors accounted for a significant 79% of the variance in DASS Depression, *R* ^2^ = 0.79, *F* (4, 116) = 86.65, *p* < 0.001. Age, sex, and oxytocin levels were not significant predictors of Depression score (**Table 3**). Both ATQ total, β = 0.783, *p* < 0.001, and cortisol level, β = 0.193, *p* < 0.001, were significant predictors, each accounting for unique variance in Depression while other variables were held constant.

**Table 3:** Summary of multiple regression analysis predicting depressive symptoms as measured by DASS Depression scores.

Predictor	*B*	β	*sr^2^*	*p*
Age	−0.003	−0.003	<0.001	0.947
Sex	0.018	0.001	<0.001	0.986
Total ATQ	0.270	0.772	0.386	<0.001
Cortisol	0.021	0.198	0.027	<0.001
Oxytocin	−0.103	−0.056	<0.001	0.227

ATQ, Automatic Thoughts Questionnaire; DASS, Depression, Anxiety and Stress Scales.

**Figure 1:**
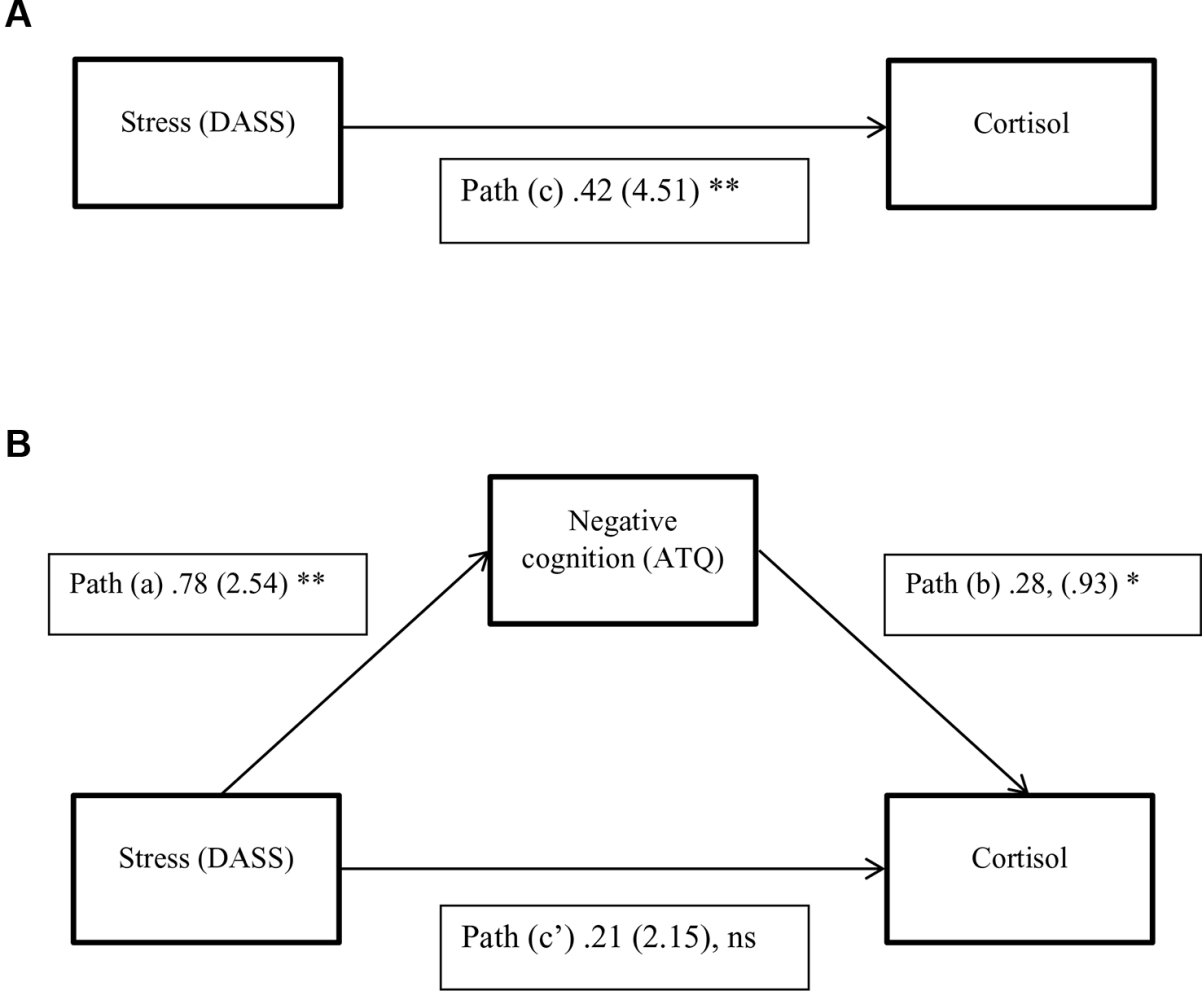
A three-variable mediation model. **(A)** The direct effect model for stress and cortisol. **(B)** The mediation model with negative cognitions as a mediator between stress and cortisol. Standardized path coefficients are shown, with corresponding unstandardized coefficients in parentheses. *P <0.05; **P < 0.01, Ns, Not significant. (*P* = .13; *N* = 121).

Results of the mediation analysis for negative cognitions in the relationship between stress and cortisol levels are shown in **Table 4** and **Figure 1**. The results indicate that the critical conditions for mediation were satisfied. 1) *Path c* was significant, *F* (2,118) = 13.56, *p* < 0.001, *R* ^2^ = 0.19, indicating that stress levels significantly predicted cortisol levels when ignoring the mediator (negative cognitions). 2) *Path a* was also significant, *F* (2,118) = 88.65, *p *< 0.001, *R* ^2^ = 0.60, indicating that stress levels significantly predicted negative cognitions. 3) When both stress and negative cognitions were entered simultaneously into the regression equation as predictors of cortisol, the overall model was significant, *F* (3,117) = 11.16, *p* < .001, *R* ^2^ = 0.22. Negative cognitions predicted cortisol (*path b*), however stress no longer predicted cortisol significantly (*path c′*). Therefore the results from all three equations were satisfied according to Baron and Kenny's (46) criteria, suggesting that negative cognitions have a mediation relationship between stress and cortisol levels. Additionally, the bootstrapping analysis with 50 000 resamples indicated that 95% CI for the indirect effect did not include zero. The indirect effect is therefore considered significant, and mediation is supported (**Table 4**). In summary, because there is evidence of mediation and the direct effect (*path c′*) is non-significant, we can infer full mediation, in other words that the relationship between stress and cortisol is fully transmitted through the mediator, negative cognitions.

**Table 4:** Mediation effects of negative cognitions on the relationship between stress levels and cortisol levels (N = 121).

Path/effect	B	SE	*t*	*p*	95% CI	
					LLCI	ULCI
*a* stress →negative cognitions	2.49	0.20	12.47	<0.00	2.09	2.88
*b* negative cognitions→ cortisol	0.98	0.43	2.31	0.02	0.14	1.83
*c* stress → cortisol	4.89	0.94	5.21	<0.00	3.03	6.75
*c'* stress → cortisol controlled for negative cognitions	2.44	1.40	1.74	0.08	0.34	5.22
(*a x b*) indirect effect stress → cortisol, with negative cognitions as a mediator †	2.45	1.29			0.12	5.20

Note: B, unstandardized coefficient; LLCI, low limit confidence interval; ULCI, upper limit confidence interval.

†Derived from 50,000 bootstrap samples.

## Discussion

To our knowledge, this is the first study to directly measure relationships between the frequency and strength of negative cognitions and either cortisol or oxytocin levels, in individuals with MDD and controls. Levels of negative thinking, stress, and psychopathology were higher in participants with MDD than in controls. Plasma cortisol levels correlated positively with all aspects of negative thinking, whereas oxytocin showed inverse correlations with aspects of negative thinking. Cortisol and negative thinking, but not oxytocin, accounted for unique variance in depressive symptoms. In addition, we found that the relationship between stress and cortisol levels depended on the extent of negative cognitions, a novel finding in research investigating psychobiological mechanisms in MDD.

The finding that participants with MDD reported experiencing both higher frequency of negative thinking and a greater strength of belief in negative cognitions than non-depressed controls was as predicted. Additionally, different domains of negative thinking were compared and participants with MDD had significantly higher levels of negative thinking across all subtypes, including personal maladjustment and desire for change, negative self-concepts and negative expectations, low self-esteem, and helplessness. This is consistent with previous research in MDD (48) and is further confirmation of the pervasive and global nature of negative thinking in depression.

Additionally, as hypothesized, negative cognitions were significantly positively correlated with cortisol levels. This was the case for all domains of negative cognitions and for both the frequency of occurrence and strength of belief in negative cognitions in MDD. Oxytocin was inversely related to thoughts of helplessness, total strength, and frequency of negative beliefs, personal maladjustment and desire for change and negative expectations. The results indicate that both cortisol and oxytocin levels are related to patterns of negative thinking; however, the relationships are somewhat broader and stronger for cortisol than oxytocin. Closer examination of the measure of negative thinking used in the current study, the ATQ, shows that few items relate to interpersonal or social aspects of depressogenic thinking. It would be of interest in future studies to assess whether interpersonally focussed negative thoughts have a stronger relationship to oxytocin levels.

We also assessed whether cognitions, cortisol, and oxytocin accounted for unique variance in depressive symptoms. Regression analysis indicated that both cognitions and cortisol accounted for unique variance in depressive symptoms after accounting for demographic factors. The findings provide further information regarding relationships between physiological aspects of depression and negative thinking, a prominent psychological feature of MDD. Hypercortisolism is one of the most consistently reported findings in research into the biology aspects of depression (e.g. 49). Additionally, glucocorticoids are neurotoxic in some circumstances and are linked to brain morphological changes seen in depression (50). While an established body of literature has linked cortisol secretion to impairments in neuropsychological functioning in MDD (51–53), previous research focussed on emotionally neutral aspects of cognition in relation to cortisol, such as concentration, memory, and executive functions. Studies investigating direct links between cortisol and negative thought content, the hallmark of MDD, were lacking, although plausible theoretical models linking the two had been proposed (4). The current results provide new information about content-specific, depressogenic aspects of cognition, and their relationship to cortisol levels. Oxytocin did not account for unique variability in depression symptoms after accounting for the other independent variables.

Additionally, we conducted a mediation analysis to test direct and indirect relationships between stress levels, negative cognitions, and cortisol levels, and to test Beck's (4) hypothesis that negative cognitions are important in the relationship between stress and cortisol. This analysis indicated that stress levels were significantly related to cortisol levels when ignoring the role of negative cognitions, and that stress levels were also significantly related to negative cognitions. However, when negative cognitions were included as a mediator in the model, negative cognitions were related to cortisol but stress was no longer a significant predictor of cortisol. The results suggest that the relationship between stress and cortisol depends on the extent of negative thinking. To our knowledge, this is the first direct empirical test of relationships between these variables, and the results appear to be consistent with Beck's (4) hypothesis that negative cognitions are important in the relationship between stress and cortisol.

Cognitive appraisals of situations have been recognized since ancient times as being of importance in determining emotional reactions to situations (54), and these assumptions form the basis of cognitive-behavioral approaches to treating emotional disorders including MDD. The current findings, however, provide firmer evidence of the potential importance of psychological processes in relation to stress and cortisol levels, an index of HPA axis activity. There are indications that cortisol responses to stress predict the course of depression trajectories (55, 56). This in turn leads to consideration of the potential for cognitive-behavioral interventions to alter HPA activity through changing cognitive interpretations of situations. Although it has been proposed that cortisol could be affected indirectly through psychotherapeutic approaches (57), there is surprisingly little research systemmatically investigating effects of psychological therapy on cortisol levels, and further research is needed.

The current results provide further information about the close links between cognitive-emotional processing and biological factors in MDD, supporting an integrated biopsychosocial model of MDD. In conjunction with previous imaging research, they suggest that cognitive appraisal may be linked to the intensity of HPA axis responses and cortisol release. Cortisol release, in turn, is related to difficulties in concentration and memory, and potential impact on the serotonergic system and depression (4). Interventions which promote realistic, rather than negative, cognitive reappraisals may minimise the impact on HPA axis activity and intercept the initiation, maintenance, and kindling of depressive episodes and their further physiological sequelae. Further research building knowledge of the interplay between cognitive factors, stress reactivity, and mood regulation may allow for more tailored interventions that match treatment approaches to aspects of HPA function (55, 56). It is also worth considering the potential for universally delivered cognitive-behavioral preventative strategies to intervene earlier between life stressors and physiogical responses and possibly prevent depression (58).

This study has several limitations which need to be considered. The mediation analysis draws on cross-sectional data and atemporal relationships do not imply causation (59). We can conclude that cognitive distortions are uniquely associated with cortisol levels, and accounting for them attenuates the relationship between stress and cortisol levels, when considering the shared relationship among all three variables (59). Additionally, symptoms and negative thinking were assessed over the preceding week; consistent with an assessment of state rather than long-term traits. Longitudinal or interventional research is needed to examine changes in hormones in relation to stress and cognitive distortions over time to confirm temporal relationships between the variables. Nevertheless, the results provide some of the first direct measures of relationships between negative cognitions and hormonal measures. We did not assess diurnal variations in cortisol between the groups in relation to the study variables, however time of collection was matched between groups. While there have been studies of diurnal cortisol levels in MDD, further research is needed to understand whether these interact with negative thinking. Additionally, future studies integrating data from brain imaging which also incorporate measures of negative thinking and cortisol would help to test and further solidify integrated models of depression. Additional research is also warranted to better understand relationships between negative thought content and other aspects of neuropsychological functioning, such as executive functioning and inhibitory processes in MDD. Future research examining relationships between oxytocin and patterns of depressogenic thinking should also include measures with greater focus on interpersonal relationships. Additionally, we did not evaluate previous stressor events or family psychiatric history in the participants. It may be informative to include such evaluations in future research.

In conclusion, we found that cortisol levels, and to a lesser extent oxytocin levels, were related to depressogenic cognitive distortions. Cortisol and cognitive distortions both accounted for unique variability in depression symptoms. Mediation analyses supported the hypothesis that the relationship between stress levels and cortisol levels depended on the extent of negative cognitive distortions. The results help to support long standing hypothetical models and have implications for potential integrated interventions for MDD incorporating cognitive and physiological approaches.

## Data Availability Statement

The datasets generated for this study are available on request to the corresponding author.

## Ethics Statement

The studies involving human participants were reviewed and approved by Joint Illawarra and Shoalhaven Local Health District and University of Wollongong Health and Medical Human Research Ethics Committee. The patients/participants provided their written informed consent to participate in this study.

## Author Contributions

ST conceived and contributed to the design of the study, acquired, analyzed and interpreted data, prepared the manuscript, and approved the final version. TL contributed to the conception and design of the study, acquired, analyzed and interpreted data, reviewed the manuscript, and approved the final version.

## Funding

This research was funded by the Faculty of Science, Medicine and Health, University of Wollongong Australia.

## Conflict of Interest

The authors declare that the research was conducted in the absence of any commercial or financial relationships that could be construed as a potential conflict of interest.
